# Factors That Shape People’s Attitudes towards the COVID-19 Pandemic in Germany—The Influence of MEDIA, Politics and Personal Characteristics

**DOI:** 10.3390/ijerph18157772

**Published:** 2021-07-22

**Authors:** Aida El-Far Cardo, Thomas Kraus, Andrea Kaifie

**Affiliations:** Institute for Occupational, Social and Environmental Medicine, Medical Faculty, RWTH Aachen University, Pauwelsstrasse 30, 52074 Aachen, Germany; aida.el-far@rwth-aachen.de (A.E.-F.C.); tkraus@ukaachen.de (T.K.)

**Keywords:** fake news, social media, survey, vaccination, SARS-CoV-2, risk perception

## Abstract

Misinformation that accompanied the current SARS-CoV-2 pandemic led to an impaired risk perception, resulting in the refusal of personal protection measures, as well as a reduced willingness to receive a vaccination. In order to identify factors that might influence people’s attitudes towards COVID-19 policies and engagement in mitigation measures, we carried out a cross-sectional study in Germany. Altogether, *n* = 808 participants completed our questionnaire concerning items on demographics, media consumption, risk perception, and trust in health authorities, as well as willingness to receive a vaccination. An overwhelming majority of our participants perceived SARS-CoV-2 as a health threat (85.7%), and almost two thirds (63.5%) mentioned they would get vaccinated against SARS-CoV-2 when a vaccination was available. A greater likelihood of vaccination intention was associated with being male (71.5% male vs. 60% female, *p* < 0.05), left-wing voting, trusting health authorities, using public media as an information source about COVID-19, and, in particular, perceiving COVID-19 as a health threat. A better understanding of factors that contribute to vaccine hesitancy is indispensable in order to eliminate doubts, increase vaccination rates, and create herd immunity, to stop further virus transmission.

## 1. Introduction

In late December 2019, Chinese authorities reported cases of pneumonia of unknown etiology in the city of Wuhan to the World Health Organization (WHO) [1]. Eventually, a novel coronavirus, called SARS-CoV-2 (severe acute respiratory syndrome coronavirus type 2), was identified as the cause. SARS-CoV-2 continued to spread steadily. On 30 January 2020, the WHO Director General declared the outbreak a “public health emergency of international concern (PHEIC)” [2]. Meanwhile, 7736 cases and 179 deaths had been confirmed in China and 107 cases of SARS-CoV-2 had been registered in 21 other countries around the globe [3]. The German federal minister of health announced at the end of February 2020, that the Coronavirus had arrived in Europe [4]. In March and April 2020, northern Italy was considered to be the epicenter of the European Coronavirus crisis [5]. Horror scenarios filled the media landscape; the capacities of Bergamo’s morgues were exhausted to such an extent that military transporters had to redistribute coffins with dead bodies to neighboring provinces for cremation [6]. Hospital staff worked at the edge of their endurance. The shortage of ventilators and intensive care beds pushed doctors to triage; i.e., decide who should be treated and kept alive and who should not [7]. In late 2020, the Robert Koch Institute (RKI), a German federal public health institution, reported new peaks of infections and deaths related to SARS-CoV-2 in Germany. The drastic rise of the 7-day-incidence from 2.5/100.000 habitants in mid-June [8] to almost 150/100.000 inhabitants in December [9] led federal states and the government to take tougher action, which resulted in a second nationwide lockdown with expanded protective measures in the middle of December 2020 [10]. Since early April 2020, protesters against the protective measures, who feared for their livelihoods, filled the streets in Germany; people who felt their basic rights were being threatened, people who were united by a deep distrust in politics and conventional medicine, people who suspected conspiracies by interest groups behind the pandemic, and also those who spread right-wing extremist ideas.

Uncertainty and mistrust in politics and scientific institutions provide the ideal breeding ground for misinformation to flourish, which can present itself in a wide variety of forms. Misinformation can range from denial, downplaying, or conspiracy theories to false and unsubstantiated claims regarding the origin of the virus and the inefficacy of cures and protective measures. The spread of these theories is a worldwide problem. The former president of the United States of America (USA), Donald Trump, constantly downplayed infection with the virus and promoted ineffective and life-threatening methods to fight it. In one alarming example, he suggested investigating a method to kill the virus by injecting disinfectant into the human body. According to Cornell University, he was described as the “largest driver of COVID-19 (coronavirus disease 2019) misinformation [...]” [11], while John Hopkins University reported that the USA were the country with the most COVID-19 cases and deaths worldwide [12,13]. Brazilian president, Jair Bolsonaro, described the coronavirus infection as “a little flu”, promoted the unproven malaria drug hydroxychloroquine as a cure, appeared in public without a mask, and refrained from introducing protective measures [14]. In Iran, a tabloid newspaper published the theory that alcohol was capable of neutralizing SARS-CoV-2. This false information led to over 5000 poisonings and 500 deaths from methanol poisoning between February and April 2020 [15]. Current societal trends, and in particular the distribution of false news by social media, make it difficult to properly address incorrect health emergency information. The start of vaccination in Germany was accompanied by a wave of disinformation that caused uncertainty among many people about getting vaccinated [16]. The WHO Director General warned of “an ‘infodemic’—an overabundance of information, some of which can be misleading or even harmful” in the face of increasing SARS-CoV-2 infection numbers [17]. Despite the precarious health crisis of international scope, what are the circumstances that allow misinformation to make its way past evidence-based scientific facts? The purpose of this paper is to examine to what extent socioeconomic factors, media consumption, information seeking behavior, and political attitudes might influence people’s attitudes towards COVID-19 policies, engagement in mitigation measures, and risk perception of the pandemic.

## 2. Theory

As the course of the COVID-19 pandemic depends on public cooperation with the recommended health protective behavior, it is becoming increasingly important to understand what encourages people to act upon it [18,19]. A large body of research has proven, that people’s risk perception is a core predictor for implementing recommended health protective-behavior [20,21,22,23,24,25]. A recent review of 149 studies on current and past global pandemics found that risk perception was the most important determinant in promoting hygiene and social distancing behavior [26]. The appraisal of risk is determined by the type of hazard, personal experiences, beliefs and attitudes, and diverse societal influences [27]. Slovic highlights in his psychometric paradigm that there is a gap between laypeople’s judgements and experts’ assessment of a hazard. While experts tend to evaluate risk on expected annual fatalities, laypeople’s risk perceptions are mainly driven by two factors: dread risk (includes the lack of control, with catastrophic potential, many fatalities) and unknown risk (unobservable, unknown, new) [28].

The public’s response to an emergency is decided by their understanding and appraisal of present risk exposure and of risk mitigation measures [27]. An accurate risk perception is fundamental to avoid exposure by adapting behavior to the new circumstances.

According to a recent metanalysis, female gender, trust in government, science and health professionals, personal knowledge of governmental strategy, being a healthcare worker, direct experience with the virus, and prosocial worldviews were positively correlated with having high risk perceptions about an infection with SARS-CoV-2 [25]. However, the risk perception might be only one among many influencing factors in implementing mitigation measures. Previous work has investigated which factors might predict adherence to recommended health protective behavior. Glöckner [22] observed in a survey in Germany that the expected long-term consequences (efficacy) of the adopted measures were significant. Evidence from the “COVID-19 Snapshot Monitoring (COSMO)” among German residents points to the role of public trust in institutions in predicting adherence to recommended health-protective behaviors and effective risk perception [29]. Dohle, Wingen [30] stated that the level of trust in politics and science was the most significant determinant for complying with health protective behavior, while the level of perceived risk was less influential. People who considered health authorities trustworthy were easier to reach through the same institutions, while distrust might build up a barrier to health communication. In addition, political attitudes have had an impact on the implementation of mitigation measures during the pandemic. A review among Western countries observed that political conservatism was related with decreased adherence to health guidance measures [31]. Investigating information seeking behavior during a health crisis is fundamental, since there is evidence that relying on social media for COVID-19 information is linked to an increased likelihood in believing misinformation [32]. As misinformation can undermine expert assessments about the virus, it presents a threat to effectively managing the pandemic. Misinformation is linked to decreased engagement in health-protective behavior [33] and willingness to vaccinate [32,34]. Brewer, Weinstein [20] stated in their research about the correlation between risk perception and vaccine uptake in the case of Lyme Disease (a vector-borne disease caused by *Borrelia burgdorferi*) that the results of the study were compatible with health behavior theories that assume that “a perceived high risk of harm should encourage people to take action to reduce their risk”. Other research reported that the most important factor was trusting the safety of the vaccine [35]. A survey in the U.S. reported that vaccination intention was significantly correlated with being male, older, identifying as a Democrat, influenza vaccination, general vaccine knowledge, rejection of vaccine conspiracies, perceived severity of COVID-19, and not relying upon social media for virus information [34].

In our investigation, we tried to evaluate the German perspective on the COVID-19 pandemic and policies. We believe that the German response to the pandemic is of particular interest, since Germany faced a growing movement against mitigation measures during the pandemic, mobilizing people on the streets across the whole country from early April 2020 on. The largest demonstration to date against the Coronavirus measures in Germany took place in Berlin at the end of August. The demonstration was initiated by the group “Querdenken 711” and was attended by an estimated 38,000 people, who demanded the abolishment of the COVID-19 restrictions. A survey conducted during a demonstration in Konstanz, a medium-sized city in Germany, observed that 93% of the respondents reported that the mitigation restrictions were exaggerated. Only 20% of the respondents stated that they trust experts when they say that SARS-CoV-2 is a health threat [36]. Accordingly, the start of German vaccination was accompanied by a wave of disinformation and had to face decreasing levels of self-reported vaccine-intentions during the course of the pandemic (79% in April/May 2020 and 48% in December 2020) [37].

## 3. Data and Methodology

This cross-sectional study was initiated in Germany. Data were collected with an online survey that was available between August and November 2020. A total of *n* = 808 persons were included in this analysis. Online questionnaires (SoSci Survey, Version 3.1.06) [38] were used, as well as paper-based questionnaires that were distributed in different medical practices. Paper-based questionnaires were used in order to reach people who were more difficult to recruit by social media campaigns, such as elderly people. Participants for the online questionnaire were recruited primarily via social networks, such as Facebook, Instagram, and WhatsApp. Respondents were able to access the electronic survey via a link that was published by the authors on different social media channels (Facebook, Instagram, Telegram) and the official website of the University Hospital RWTH Aachen. Other links to access the survey were distributed by the authors and authors’ friends and relatives via Whatsapp. Our results are therefore based upon a convenience sample that is not representative of the German population. Most respondents came from the following German federal states: North-Rhine Westphalia (38.9%), Rhineland Palatinate (22.1%), and Saarland (14.7%). Ethical approval for the study was obtained from the local Human Research Ethics Committee of the RWTH Aachen University.

### 3.1. Survey Structure

The data were collected with a 32-item questionnaire. The questionnaire contained questions and statements about the following topics:

1. Demographic characteristics: age, residency, level of education, employment.
2. Political views.
3. Media consumption: social networks and messenger services, information seeking behavior (television, print media, radio). Public media in Germany is an independent source of information without any influence of state or private sector. Public media is financed through broadcasting fees by the German population and includes television, radio, and social media channels. In our analyses we included public media TV channels (e.g., ARD, ZDF) under the term “public media”, since they are the most important source of news in Germany.
4. Knowledge about SARS-CoV-2 and personal experience with SARS-CoV-2.
5. Risk perception: we considered that respondents perceived the virus as a threat to health when they answered to the statement “I am concerned that an infection with the SARS-CoV-2 could damage my health or a relative’s health” with “agree” or “strongly agree”.
6. Satisfaction with public health education about the pandemic by certain institutions.
7. Assessment of the necessity of SARS-CoV-2-specific health-protective measures and willingness to get vaccinated.
8. Implementation of SARS-CoV-2-specific health-protective measures.
9. Trust in health authorities and governmental institutions.
10. Attitudes towards vaccinations in general.
11. Attitudes towards alternative medicine in general.

Certain views and attitudes, such as risk perception, satisfaction with public health education, protective measures, or trust in health authorities were queried using a four-point Likert scale.

### 3.2. Statistical Analysis

Survey data were collected and analyzed with SAS Software (SAS 7.1, SAS Institute Inc., Cary, NC, USA). Descriptive analyses of categorical variables, such as demographic characteristics, media consumption, knowledge and personal experience with SARS-CoV-2, and implementation of protective measures were carried out. Demographic characteristics, political attitudes, use of social media, information seeking behavior, and trust in health authorities were related to the risk perception of SARS-CoV-2 and willingness to get vaccinated. All statistical tests were two-sided, and *p* < 0.05 was used as the level of significance.

## 4. Results

Our sample consisted of *n* = 808 participants (female = 68.9%; mean age = 42.9 years; SD = 10.1). The educational level was high, 27.4% of the respondents had a university degree and 18.3% were students. A majority of the participants (68.7%, *n* = 564) sourced information about the pandemic through public media. More details about the characteristics of the cohort can be found in Table 1. We observed an increase of risk perception in our cohort from the beginning of the COVID-19 pandemic in comparison to the time of the survey. A high proportion of our respondents (85.7%; *n* = 634) classified SARS-CoV-2 as dangerous at the time of data collection (Table 2), while only 34.6% (*n* = 270) indicated that they were concerned about their own or their relative’s health when the pandemic was declared by the WHO in March 2020. Interestingly, only 56.5% (*n* = 417) were worried about being infected with the virus. A large majority (88.9%; *n* = 663) estimated the use of face masks as “very necessary” or “necessary”. About 63.5% (*n* = 478) declared an intention to receive the COVID-19 vaccine. A clear majority of our participants trusted health authorities such as the WHO/RKI (86.7%; *n* = 621)) or the Federal Ministry of Health (BMG) (87.3%; *n* = 616)). Most indicated their information source for COVID-19 was public media (68.7%; *n* = 564) followed by websites from health authorities (53.1%; *n* = 422). Regarding health knowledge, 70.8% (*n* = 548) of the respondents were able to answer all of our questions about COVID-19 correctly. In addition, 1.9% (*n* = 15) of our participants had been infected with SARS-CoV-2, while 30,1% (*n* = 232) reported having relatives who had been infected.

### 4.1. Risk Perception

**Figure 1:**
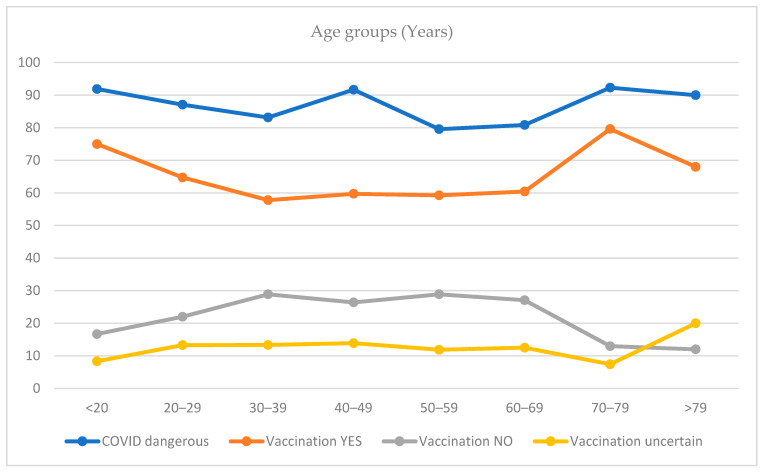
Risk perception and vaccination intention among different age groups.

The majority (85.7%; *n* = 634) of the participants classified COVID-19 as a significant health risk. With regard to demographics, women were more likely to perceive SARS-CoV-2 as a health threat (86.9%; *n* = 437) in comparison to men (83.2%; *n* = 193) (*p* < 0.05); whereas education did not significantly affect the risk perception (*p* = 0.63). Comparing age groups, the highest risk perception could be observed among people between 70–79 years (92.3%; *n* = 48), followed by people of 19 years and younger (91.9%; *n* = 34) and people between 40–49 years old (91.7%; *n* = 66). People aged 50–59 had the slightest risk perception among all age groups with 79.6% (*n* = 105) (Figure 1).

### 4.2. Health Protective Behavior

People’s engagement in health protective measures, such as the use of face masks, physical distancing, or hand hygiene was related to the perceived disease risk. From a total of 617 people who considered COVID-19 as a threat to health, 95.5% (*n* = 589) indicated complying with at least three recommended COVID-19 mitigation measures. Participants who indicated a lower risk perception (*n* = 104) reported significantly less approval of recommended mitigation measures (69.2%; *n* = 72) (*p* < 0.01).

### 4.3. Trust in Institutions

We found significant differences in the perceived trustworthiness of health authorities for respondents who classified SARS-CoV-2 as dangerous compared to participants who classified SARS-CoV-2 as less dangerous, *p* < 0.01 (Table 3). Thus, 92% (*n* = 573) of the respondents that perceived SARS-CoV-2 as a health threat indicated they trusted the German Federal Ministry of Health, while it was 95.8% (*n* = 573) for the RKI/WHO. Among respondents who did not perceive SARS-CoV-2 as a risk to health, only 59.8% trusted both the RKI/WHO, as well as the German Federal Ministry of Health.

### 4.4. Media

People seeking information about COVID-19 through websites from health authorities (*n* = 360; 89.6%) (*p* < 0.01), public media (*n* = 457; 88.4%) (*p* < 0.01), and daily journals (*n* = 213; 89.9%) (*p* < 0.05) showed a significantly higher risk perception. Sourcing COVID-19 information through social media (*n* = 127; 81.4%) (*p* = 0.09) did not result in a significant difference regarding risk perception, although there was a trend observable (Table 3). However, a detailed investigation of different social media channels showed that 21.7% of the participants that used Telegram and 16.5% of participants using Facebook as a source of information for SARS-CoV-2 did not perceive the virus as a health threat.

### 4.5. Political Attitude

About 89.7% (*n* = 376) of the participants who were left-wing voters indicated that they considered SARS-CoV-2 as a health threat, compared to 87.6% (*n* = 148) among conservative-liberal voters. We found a strong negative association between voting for right-wing populism and risk perception (Table 3). Only 38.5% (*n* = 5) of the right-wing populism voters stated they perceived SARS-CoV-2 as a threat to health, *p* < 0.01. We also observed a negative association between COVID-19 risk perception and non-voting. Moreover, 69.1% (*n* = 56) of the participants that reported they would not vote, perceived the virus as risk to health compared to 87.4% (*n* = 548) of the voters, *p* < 0.01. While 53.0% (*n* = 44) of non-voters reported they would not get vaccinated against SARS-CoV-2, only 20.4% (*n* = 130) of voters would reject a vaccination, *p* < 0.01. Among voters, 20.6% (*n* = 36) of conservative-liberal voters, 17.8% (*n* = 75) of left-wing voters, and 64.3% (*n* = 9) of right-wing populism voters reported they would not get vaccinated against SARS-CoV-2, *p* < 0.01.

### 4.6. Own Experience

We could not observe any statistically significant differences regarding SARS-CoV-2 risk perception for people who were infected themselves (*p* = 0.4) or had infected relatives (*p* = 0.07), although the association between infected relatives and their own risk perception showed a trend.

### 4.7. Vaccination Intention

**Figure 2:**
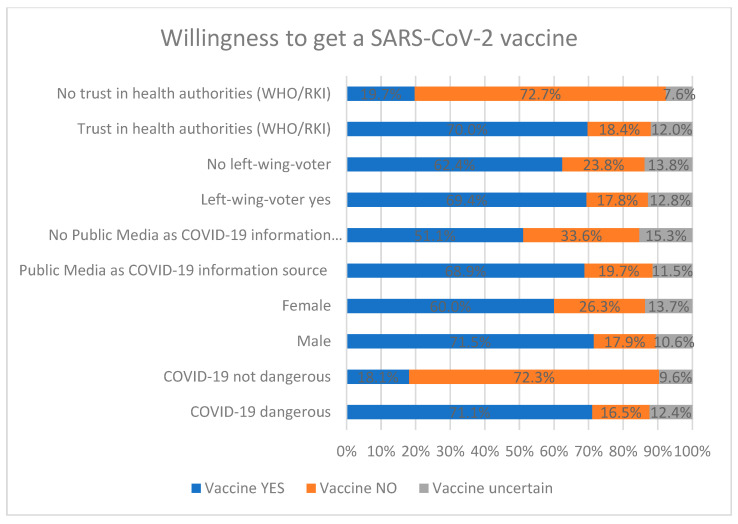
Determinants of willingness to get vaccinated against SARS-CoV-2.

Among all participants 38.9% (*n* = 293) strongly agreed, 24.6% (*n* = 185) agreed, 10.9% (*n* = 82) disagreed, 13% (*n* = 98) strongly disagreed, and 12.6% (*n* = 95) were uncertain about receiving a vaccination against SARS-CoV-2, when available. A greater likelihood of vaccination intention was associated with being male (71.5% male vs. 60% female, *p* < 0.05), left-wing voting, trusting health authorities such as the Federal Ministry of Health, WHO or RKI, using public media as an information source about COVID-19, and in particular perceiving COVID-19 as a health threat (Figure 2). We observed no statistically significant differences in the intention to get vaccinated between healthcare workers (68.4%; *n* = 104) and people not working in the healthcare sector (62.2%; *n* = 375), *p* = 0.36. Further factors that were negatively associated with the intention to get vaccinated were using social media as an information source about SARS-CoV-2 (*p* < 0.01), and the use of Facebook (*p* < 0.05) or Telegram (*p* < 0.05) in general. However, using Twitter was not significantly associated with adverse vaccination behavior (*p* = 0.56).

## 5. Discussion

The current study sheds light on the German public response to the COVID-19 pandemic, between the end of the first lockdown and the start of a second lockdown due to a second wave of SARS-CoV-2 infections (August–November 2020). Our research questions focused on the influence of a wide variety of determinants on risk perception and vaccination intention and how risk perception might shape engagement with the recommended health-protective behaviors and vaccination intention. In accordance with previous published data, an overwhelming majority of our participants perceived SARS-CoV-2 as a health threat [22,25,39] Almost two thirds mentioned they would get vaccinated against SARS-CoV-2 when a vaccination is available. People aged 70–79 years had the highest values for both, SARS-CoV-2 risk perception, as well as the intention to get vaccinated, which is not surprising considering since aged people are more likely to develop a severe disease course. Women were more likely to perceive COVID-19 as a threat to health, even though there were higher fatality rates among men [40]. Nevertheless, men mentioned they were more likely to get vaccinated once a vaccine becomes available, which was in a line with recent research from Ruiz and Bell [34]. Against our expectations, we did not find a significant difference for people who had direct experience with the virus through infected relatives or personal infection, which was in contrast to findings from Dryhurst, Schneider [25]. In addition, our results did not show significantly higher vaccination intention rates for healthcare workers. A survey conducted in mid-December 2020 by the “Germany Society for Intensive Care and Emergency Medicine (DGIIN)” among healthcare workers in Germany observed that 75% of the physicians reported an intention to get vaccinated, while only 50% of the nursing staff did so. Many nursing staff stated being concerned about side-effects and/or long-term damages related to the vaccine [41].

Results from our research also suggested that being politically left-orientated, trusting health authorities, and seeking information about the virus from public media or websites of health authorities were positively associated with perceiving the virus as a threat to health and also with having higher vaccination intentions. This is in accordance with a prior study from the U.S. finding that voters of the Democratic Party had higher vaccination intentions compared to voters from independent parties or the Republican party [34]. Our investigation found slightly lower vaccine acceptance and risk perception among conservative-liberal voters, which was not significant compared to left-wing voters. Non-voters and right-wing populism voters mentioned a significantly reduced intention to get vaccinated against COVID-19 and had a significantly lower risk perception as well. The significantly lower risk perception and vaccination intention of non-voters might be a statement of a lack of trust in politics. This assumption is strengthened by the fact that non-voters significantly more often stated to distrust federal institutions and health authorities. Previous research investigated how trust in politics, science, and health authorities might influence people’s risk perception and handling of the pandemic, finding that those who trusted public institutions were more likely to comply with the recommended behavior [29,30,31,42]. In accordance with the “Weekly COVID-19 Snapshot Monitoring (COSMO)” among German residents, an overwhelming majority of our participants stated they trusted health authorities [29]. We observed, congruent with Eitze, Felgendreff [29], a positive relationship between perceived threat and trust in institutions in our participants. Our results also suggest that people who trust health authorities are significantly more willing to receive the COVID-19- vaccine, which was consistent with previous findings [34]. A study based on survey data from 25 European countries described that countries with less institutional trust had higher mortality rates [43]. This highlights that authorities and experts that provide information about a health crisis have to be considered as trustworthy by the public in order to provoke a behavioral shift and protect from illness [44]. The credibility of such authorities can be undermined by misinformation [45]. Information seeking behavior affected people’s attitudes and perceptions of the pandemic [42,46]. Public media was the most popular source of information in our cohort, followed by official websites from health authorities. We observed that both information sources were related with a high risk-perception and vaccination intention; whereas, consistent with Ruiz and Bell [34], reliance on social media for COVID-19 information negatively affected vaccination intention. However, it should be noted that information seeking about COVID-19 and risk perception may reciprocally interact with each other [47]. Previous research showed that health-related misleading and false information is often shared through social media [33,48,49]. Political conservatism [32], relying on social media for COVID-19 information [32,33], and lack in trust in health authorities [50] are clearly linked to an increased susceptibility to misinformation. Existing studies proved that misinformation decreases the implementation of the recommended mitigation measures [32,33] and intention to get vaccinated [32]. Therefore, the spread of misinformation is definitely a serious public health threat [51]. While our study did not investigate the belief of false information about SARS-CoV-2, the above mentioned theories offer a possible explanation why people who seek information about COVID-19 through social media were significantly less willing to be vaccinated against SARS-CoV-2. However, we did not observe a significant association between using social media as an information source about SARS-CoV-2 and reduced SARS-CoV-2 risk perception in our analysis. In accordance with previous research, we observed that individuals who perceived that COVID-19 posed a greater risk to health were more likely to comply with health-protection measures [22,25,31,39,47]. This observation is not unique to the Coronavirus pandemic but has been proven during earlier health emergencies. Those findings indicate that an accurate threat-perception is of crucial importance, as the extent and speed of the spread of the virus depend on adherence to the recommended protective behavior [52]. The level of perceived disease risk might be only one among several influencing factors for receiving a vaccination [20,34,53]. A better understanding of factors that contribute to vaccine hesitancy is indispensable in order to eliminate doubts, increase vaccination rates, and create herd immunity to stop further virus transmission.

This is only feasible, if public health campaigns addressing risk perception and vaccination hesitancy identify specific target groups. For example, campaigns to increase vaccination rates should take into consideration that women were less willing to get vaccinated and try to understand where this vaccination hesitancy comes from. From our study results we could also identify non-voters as a specific group that should be addressed in order to raise vaccination rates. It might be challenging to reach groups that distrust health authorities through official health campaigns, as they might reject expert assessments and therefore health information from official institutions. However, health authorities are responsible for raising awareness and sharing knowledge about the pandemic among the whole population, even for those who are willing to reject all recommended measures. In addition, health authorities should identify channels via which they can reach targets, in particular those groups that do not use public media or health authorities’ websites as a source of information about the Coronavirus. As already implemented in part, social media channels could indicate if an information source is trustworthy or not and help to reduce the amount of misinformation around the virus and the vaccine.

As a first step, policymakers and public health authorities need to understand what causes this lack of trust in their assessments, try to restore it, and finally enable effective health communication. Our findings specifically provide a list of potential factors that official health campaigns may address when trying to improve engagement in health-protective behavior and vaccination.

### Limitations

The majority of the participants were recruited via social media and the network of the authors. We used a convenience sample that is not representative of the general population of Germany. Data were collected between August and November 2020. All our findings relate exclusively to this period of the survey. Due to a lack of temporal data, we cannot draw any conclusions on the actual situation in Germany. Only *n* = 17 participants described themselves as right-wing populism voters. Therefore, general conclusions on right-wing populism voters are only possible to a limited extent. Data were exclusively collected via self-reports, so that we cannot exclude social desirability bias.

## 6. Conclusions

In conclusion, a large majority of our respondents had a high risk perception and high vaccination intention. We observed that a higher perceived risk strengthened the implementation of health-protective measures and the intention to get vaccinated. Both, a high risk perception and vaccination intention were significantly related with voting left, trusting health authorities, and seeking information through public media and health authority websites. Additionally, vaccination hesitancy was related with the female gender and sourcing COVID-19 information through social media. Since we do not yet have a medical cure, the course of the pandemic depends on the extent to which the population implements preventive measures. These include health-protective behavior and vaccination intention, both of which have been shown to be closely related to risk perception.

Another important finding was the strong association between non-voting and vaccination hesitancy. As non-voting might be a symptom of lacking trust in health authorities, we underline the importance of building up transparent communication between policymakers and public.

## Figures and Tables

**Table 1:** Demographic Characteristics.

Characteristics	Frequency (*n*)	Percentage (%)
Gender	*n* = 808	
Female	557	68.9
Male	250	30.9
Divers	1	0.1
Political attitude	*n* = 674	
Left-wing	449	66.6
Conservative-liberal	118	17.5
Right-wing populism	17	2.5
Non-voter	90	13.4
Level of education	*n* = 791	
Scholar	24	3.0
Student	145	18.3
General secondary school	83	10.5
Intermediate secondary school	114	14.4
Technical college	55	7.0
Grammar school	92	11.6
University of Applied Sciences	61	7.7
University degree	217	27.4
Working status	*n* = 801	
Working	481	60.1
Seeking work	46	5.7
Pension	146	18.2
No, other	128	16.0
Working sector	*n* = 591	
Education	77	9.5
Hospitality industry	19	2.3
Health industry	166	20.4
Marketing	53	6.5
Arts, wellness, leisure	29	3.6
Public administration	63	5.3
Manufacturing	35	4.3
Other	149	18.4
COVID-19 Information Sources	*n* = 795	
Public media	564	68.7
Private TV-channels	101	12.7
Daily Journals	254	32.0
Online news	276	34.7
Family and relatives	192	24.2
Search engines	228	28.7
Health authorities	422	53.1
Social media	166	20.9
Celebrities	5	0.6
Radio	252	31.7
Podcasts	107	13.5

**Table 2:** Risk perception, vaccination intention, trust in health authorities, and health protective measures among all participants.

**Risk perception**	When the global pandemic was declared by the WHO in March 2020, what was your attitude towards the Coronavirus?		
	I was worried about my health or the health of my relatives	270 (34.6%)	
	I didn’t feel threated	511 (65.4%)	
	If your perception changed since then, what did change?		
	I currently perceive the Coronavirus as less dangerous	142 (38.1%)	
	I currently perceive the Coronavirus as more dangerous	231 (61.9%)	
	Please indicate whether you agree on the following statements or not	Strongly agree or agree	Strongly disagree or disagree
	I am worried that I may get infected with the virus	417 (56.5%)	321 (43.5%)
	I am worried that an infection with the Coronavirus could threat my health or the health of my relatives	634 (85.7%)	106 (14.3%)
	I think that the Coronavirus gets too much attention	203 (27.7%)	530 (72.3%)
**Vaccination** Intention *	As soon as a Coronavirus vaccine is available, I would get vaccinated with the new vaccine	478 (63.5%)	180 (23.9%)
**Trust in Institutions/** **persons for reliable medical information**	German Federal Ministry of Health (BMG)	616 (87.3%)	90 (12.7%)
	World health organization/Robert Koch institute (WHO/RKI)	621 (86.7%)	68 (9.5%)
	Family physicians	598 (88.6%)	77 (11.4%)
	Family/friends	334 (51.1%)	320 (48.9%)
	Own research	496 (77.9%)	141 (22.1%)
**Health Protective Measures**	Which SARS-CoV-2 health protective measures do you apply in your daily life?		
	Wearing face mask	690 (92.7%)	
	Regular hand-disinfection	511 (68.9%)	
	Washing hands for 20 s	584 (78.5%)	
	Staying at least 1.50 m apart from anyone outside of the own household	644 (86.6%)	
	Staying home when feeling sick	580 (78.0%)	
	None	5 (0.7%)	

* *n* = 95 (12.6%) were uncertain about receiving a vaccination.

**Table 3:** Influencing factors on risk perception and vaccination intention.

	SARS-CoV-2 as a Health Threat *n* (%)	*p*-Value *	Vaccination Yes *n* (%)	Vaccination No *n* (%)	Vaccination Uncertain *n* (%)	*p*-Value *
Gender						
Male	193 (83.2)	<0.05	168 (71.5)	42 (17.9)	25 (10.6)	
Female	437 (86.9)		308 (60.0)	135 (26.3)	70 (13.7)	<0.05
Diverse	0(0)		0 (0)	1 (100)	0 (0)	
Political Ideology						
Conservative-liberal	148 (87.6)	<0.01	114 (65.1)	36 (20.6)	25 (14.3)	
Left	376 (89.7)		293 (69.4)	75 (17.8)	54 (12.8)	<0.01
Right-voter	5 (38.5)		4 (28.6)	9 (64.3)	1 (7.1)	
Non-voter ^#^	56 (69.1)	<0.01	29 (34.9)	44 (53.0)	10 (12.1)	<0.01
COVID Information Sources						
Public media yes	457 (88.4)	<0.01	361 (68.9)	103 (19.7)	60 (11.5)	
Public media no	177 (79.4)		117 (51.1)	77 (33.6)	35 (15.3)	<0.01
Daily journal yes	213 (89.9)		176 (71.8)	41 (16.7)	28 (11.4)	
Daily journal no	421 (83.7)	<0.05	302 (59.5)	139 (27.4)	67 (13.2)	<0.01
Online news yes	229 (87.7)		173 (65.5)	48 (18.2)	43 (16.3)	
Online news no	405 (84.6)	0.24	305 (62.4)	132 (27.0)	52 (10.6)	<0.01
Health authorities yes	360 (89.6)		276 (67.8)	84 (20.6)	47 (11.6)	
Health authorities no	274 (81.1)	<0.01	202 (58.4)	96 (27.8)	48 (13.9)	<0.05
Social media yes	127 (81.4)		82 (52.2)	49 (31.2)	26 (16.6)	
Social media no	507(86.8)	0.09	396 (66.4)	131 (22.0)	69 (12.0)	<0.01
Trust in Institutions						
BMG yes	543 (89.9)		428 (70.0)	111 (18.2)	72 (11.8)	
BMG no	47 (53.4)	<0.01	26 (29.2)	55 (61.8)	8 (9.0)	<0.01
WHO/RKI yes	573 (90.4)		448 (69.7)	118 (18.4)	77 (12.0)	
WHO/RKI no	25 (37.9)	<0.01	13 (19.7)	48 (72.7)	5 (7.6)	<0.01
Family physician yes	506 (86.9)		398 (67.0)	124 (20.9)	72 (12.1)	
Family physician no	62 (81.6)	<0.05	39 (53.4)	27 (37.0)	7 (9.6)	<0.01
Use of social networks and messenger services						
Telegram yes	119 (78.3)		90 (58.8)	48 (31.4)	15 (9.8)	
Telegram no	515 (87.6)	<0.01	388 (64.7)	132 (22.0)	80 (13.3)	<0.05
Facebook yes	353 (83.7)		256 (60.4)	118 (27.8)	50 (11.8)	
Facebook no	281 (88.4)	0.07	222 (67.5)	62 (18.8)	45 (13.7)	<0.05
Twitter yes	72 (87.8)		54 (64.3)	17 (20.2)	13 (15.5)	
Twitter no	562 (85.4)	0.56	424 (63.4)	163 (24.4)	82 (12.3)	0.56

* chi-square test; ^#^ non-voter vs. voter

## Data Availability

Data can be made available upon request.
